# Level of serum soluble lumican and risks of perioperative complications in patients receiving aortic surgery

**DOI:** 10.1371/journal.pone.0247340

**Published:** 2021-03-04

**Authors:** Ming-En Hsu, Yu-Ting Cheng, Chih-Hsiang Chang, Yi‐Hsin Chan, Victor Chien-Chia Wu, Kuo-Chun Hung, Chia-Pin Lin, Kuo-Sheng Liu, Pao-Hsien Chu, Shao-Wei Chen

**Affiliations:** 1 Department of Medicine, Chang Gung University, Taoyuan City, Taiwan; 2 Division of Thoracic and Cardiovascular Surgery, Department of Surgery, Chang Gung Memorial Hospital, Linkou Medical Center, Chang Gung University, Taoyuan City, Taiwan; 3 Kidney Research Center, Division of Nephrology, Chang Gung Memorial Hospital, Linkou Branch, Taoyuan City, Taiwan; 4 Department of Cardiology, Chang Gung Memorial Hospital, Linkou Medical Center, Chang Gung University, Taoyuan City, Taiwan; 5 Center for Big Data Analytics and Statistics, Chang Gung Memorial Hospital, Linkou Medical Center, Taoyuan City, Taiwan; National Yang-Ming University, TAIWAN

## Abstract

**Objective:**

Several serum biomarkers have been investigated for their potential as diagnostic tools in aortic disease; however, no study has investigated the association between serum biomarkers and outcomes after aortic surgery. This study explored the predictive ability of serum soluble lumican in postoperative outcomes after aortic surgery.

**Methods:**

In total, 58 patients receiving aortic surgery for aortic dissection or aneurysm at Linkou Chang Gung Memorial Hospital in Taiwan in December 2011–September 2018 were enrolled. Blood samples were collected immediately upon patients’ arrival in the intensive care unit after aortic surgery. The diagnostic properties of soluble lumican levels were assessed by performing receiver operating characteristic (ROC) curve analysis. The confidence interval (CI) of the area under the ROC curve (AUC) was measured using DeLong’s nonparametric method and the optimal cutoff was determined using the Youden index.

**Results:**

The serum soluble lumican level distinguished prolonged ventilation (AUC, 73.5%; 95% CI, 57.7%–89.3%) and hospital stay for >30 days (AUC, 78.2%; 95% CI, 61.6%–94.7%). The optimal cutoffs of prolonged ventilation and hospital stay for >30 days were 1.547 and 5.992 ng/mL, respectively. The sensitivity and specificity were respectively 100% (95% CI, 71.5%–100%) and 40.4% (95% CI, 26.4%–55.7%) for prolonged ventilation and 58% (95% 27.7%–84.8%) and 91.3% (95% CI, 79.2%–97.6%) for hospital stay for >30 days.

**Conclusions:**

The serum soluble lumican level can be a potential prognostic factor for predicting poor postoperative outcomes after aortic surgery. However, more studies are warranted in the future.

## Introduction

Because aortic diseases are challenging medical conditions [1–4], prompt surgical interventions are often required that are accompanied by several complications, such as respiratory dysfunction requiring mechanical ventilation support, acute kidney injury, hemorrhage requiring re-exploration, and prolonged intensive care unit (ICU) and hospital stay [5–7]. These complications can cause mortality or influence postoperative quality of life. For example, stroke leads to mortality or long-term care, and bleeding causes infection, blood transfusion, and even re-exploration. Prolonged mechanical ventilation leads to lung injury, tracheostomy, respiratory failure requiring long-term respiratory care, and higher mortality rates [8]. Therefore, useful tools that predict complications are crucial to provide proper and precise intervention to improve patient outcomes. A serum biomarker is a relatively noninvasive and easily accessible tool. Development of promising serum biomarkers that predict complications may substantially improve prognosis in the future.

To date, many studies have investigated biomarkers of aortic diseases [9], such as smooth muscle myosin heavy chain [10,11], creatine kinase-BB isozyme [12], calponin [13], matrix metalloproteinases [14,15], transforming growth factor β [16,17], and D-dimer [18,19]. Lumican, a small leucine-rich proteoglycan, is a component of the extracellular matrix constituting numerous body tissues, such as aortic smooth muscle cells [20] and coronary atherosclerosis tissues [21], and can play multiple roles such as cell migration, proliferation, and collagen fibrogenesis [22]. Previous studies [23,24] have demonstrated that lumican can be a promising serum biomarker for diagnosing and predicting the severity of acute aortic dissection. Another study [25] demonstrated that lumican may predict future aneurysms in patients with bicuspid aortic valve undergoing aortic valve surgery. Our animal study [22] suggested that lumican promotes lung injury. We hypothesized that a higher serum soluble lumican level is associated with aortic diseases of greater severity; therefore, additional complications, especially respiratory related, may arise after aortic surgery. According to our previous study [26], the oxygenation index (PaO_2_/FiO_2_) in acute respiratory distress syndrome group improved on the fourth postoperative day. The primary outcome of this study was prolonged mechanical ventilation >72 hours. Hence, this study investigated whether the serum soluble lumican level can be a promising biomarker for predicting postoperative outcomes in aortic surgery.

## Materials and methods

### Study population and data collection

A total of 58 patients who underwent aortic surgery for aortic dissection or aneurysm at Linkou Chang Gung Memorial Hospital in Taiwan from December 2011 to September 2018 were enrolled in this cohort study. The patients provided written informed consent, and blood samples were collected immediately upon patient arrival at the ICU after aortic surgery. The soluble lumican level was determined using the enzyme-linked immunosorbent assay (ELISA). The demographic data, surgery details, and in-hospital outcomes were recorded through careful chart reviews. Medical records were last accessed on November 16, 2019. This study was approved by the Institutional Review Board of Chang Gung Memorial Hospital (103-1993B).

### Comorbidities and outcomes

Ventilation time was defined as the duration from ICU arrival after surgery to extubation. A ventilation time of >72 hours was defined as prolonged ventilation and also termed as the primary outcome of this study. Oxygenation indices (PaO_2_/FiO_2_) were determined by performing arterial blood gas analysis, and only the lowest values were recorded per day. New onset stroke was suspected by postoperative clinical symptoms and confirmed through computed tomography and consultation with a neurologist. Patients who underwent a second surgery for bleeding were classified under re-exploration for bleeding, and those who required dialysis for acute kidney injury were classified under *de novo* dialysis. Sepsis was diagnosed on the basis of clinical symptoms and positive studies of blood cultures. All the patients were followed-up until discharge.

### Measurement of the soluble lumican level

Blood samples were collected in heparinized tubes via indwelling arterial catheters immediately when the patients arrived in the ICU after aortic surgery and centrifuged at 1000 × *g* for 10 minutes. Then, the clarified supernatants were aliquoted and stored at −80°C until further analysis. The soluble lumican level was determined using ELISA according to the manufacturer’s protocol (CSB-E09797h, Cusabio Biotech Co., Ltd., Wuhan, China). ELISA readers were used to detect signals from 96-well plates.

### Statistical analysis

The data of patient characteristics are presented as numbers and frequencies for categorical variables, means ± standard deviations for continuous variables with normal distribution, and medians (25^th^ and 75^th^ percentiles) for continuous variables with skewed distribution. The diagnostic properties of the soluble lumican level in discriminating binary in-hospital outcomes were assessed by performing the receiver operating characteristic (ROC) curve analysis. The confidence interval of the area under the ROC curve (AUC) was constructed using the nonparametric method. The optimal cutoff of the soluble lumican level was determined using the Youden index. All tests were two-tailed, and *P* < 0.05 was considered statistically significant. No adjustment in multiple testing (multiplicity) was made in this study. Data analyses were performed using SPSS 25 (IBM SPSS Inc, Chicago, Illinois).

## Results

### Baseline data and in-hospital outcomes

The demographic data, surgery details, and in-hospital outcomes of the 58 patients are listed in **Table 1**. The average age was 55.9 years (standard deviation: 13.6 years), and a quarter of the patients were women (27.6%; 16/58). Approximately three-quarters (74.1%; 43/58) of the cohort had hypertension and one-tenth were diagnosed as having heart failure and chronic kidney disease. Overall, 39.7% underwent ascending aorta replacement; 46.6%, 15.5%, and 20.7% underwent aortic extension surgeries for aortic arch replacement, aortic root replacement, and elephant trunk, respectively. Only 3 (5.2%) and 11 (19%) patients respectively underwent concomitant coronary artery bypass graft (CABG) and concomitant valve surgeries, respectively. The median bypass time, clamp time, and arrest time were 225.5, 145.5, and 37.0 minutes, respectively. The median ventilation time was 19.5 hours. The median ICU stay was 4 days, and the median hospital stay was 15.5 days. The in-hospital mortality rate was 3.4% (2/58). The median serum lumican level of the study population was 4.6 ng/mL (**Table 1**).

**Table 1:** Demographic, preoperative, and perioperative data of study patients (*N* = 58).

Variable	*N* (percentage) or mean ± standard deviation or median [Q1, Q3]
Demographics	
Age, years	55.9 ± 13.6
Female	16 (27.6)
Body mass index (kg/m^2^)	27.4 ± 5.6
Smoking	16 (27.6)
Comorbidity	
Heart failure	8 (13.8)
Diabetes mellitus	2 (3.4)
Hypertension	43 (74.1)
Old stroke	4 (6.9)
COPD	1 (1.7)
Chronic kidney disease	6 (10.3)
Surgical type	
Ascending aorta replacement	23 (39.7)
Aortic arch replacement	27 (46.6)
Aortic root replacement (Bentall operation)	9 (15.5)
Elephant trunk	12 (20.7)
Additional surgery	
CABG	3 (5.2)
Valve surgery	11 (19.0)
Intra-operative data, min	
Bypass time, min	225.5 [175.0, 282.0]
Clamp time, min	145.5 [130.0, 182.0]
Arrest time, min	37.0 [0.0, 50.0]
HTK solution	54 (93.1)
Preoperative status	
Hemopericardium	14 (24.1)
Intubation	2 (3.4)
Neurological defect	8 (13.8)
Moderate or severe aortic regurgitation	23 (39.7)
In-hospital outcome	
Ventilation time, hours	19.5 [8.0, 53.5]
Prolonged ventilation (≥72 hrs.)	11 (19.0)
Cardiogenic shock and need MCS	1 (1.7)
New onset stroke	13 (22.4)
Re-exploration for bleeding	13 (22.4)
ICU stay, days	4.0 [2.0, 7.0]
ICU stay ≥7 days	17 (29.3)
Hospital stay, days	15.5 [9.0, 24.0]
Hospital stay ≥30 days	12 (20.7)
*de novo* dialysis	5 (8.6)
Sepsis	2 (3.4)
Deep wound infection	2 (3.4)
Mortality	2 (3.4)
Lumican, ng/ml	4.6 [1.3, 4.8]

CABG, coronary artery bypass graft; COPD, chronic obstructive pulmonary disease; ICU, intensive care unit; MCS, mechanical circulation support; Q, quartile.

### Diagnostic properties of the soluble lumican level in discriminating in-hospital complications

**Fig 1:**
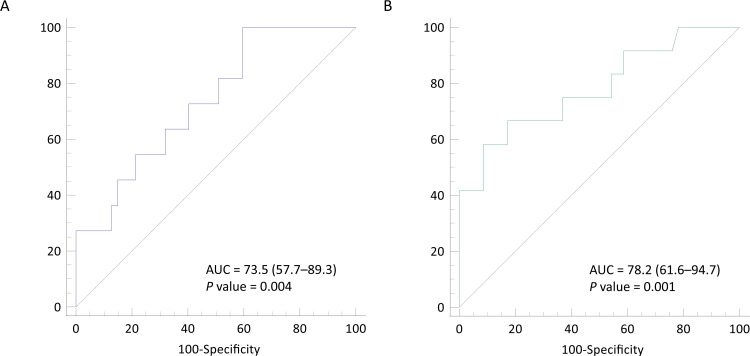
The receiver operating characteristic curves of serum lumican in discriminating prolonged ventilation (A) and hospital stay ≥30 days (B). Abbreviation: AUC, area under curve.

The diagnostic properties of the soluble lumican level in discriminating different in-hospital outcomes are listed in **Table 2**. The results revealed that the soluble lumican level could distinguish prolonged ventilation (AUC, 73.5%; 95% confidence interval [CI], 57.7%–89.3%) and hospital stay over 30 days (AUC, 78.2%; 95% CI, 61.6%–94.7%) (**Fig 1**). However, the discrimination ability of the soluble lumican level for stroke, re-exploration for bleeding, new onset dialysis, and prolonged ICU stay (≥7 days) was not statistically significant. The optimal cutoff level for prolonged ventilation was 1.547 ng/mL, and the sensitivity and specificity were 100% (95% CI, 71.5%–100%) and 40.4% (95% CI, 26.4%–55.7%), respectively. Eighteen patients exhibited lumican levels lower than 1.547 ng/mL. Regarding hospital stay over 30 days, the optimal cutoff level was 5.992 ng/mL, and the sensitivity and specificity were 58% (95% 27.7%–84.8%) and 91.3% (95% CI, 79.2%–97.6%), respectively.

**Table 2:** Diagnostic properties of serum lumican in discriminating different in-hospital outcomes.

Outcome	AUC (95% CI)	*P* value	Optimal cutoff #	Sensitivity (95% CI)	Specificity (95% CI)
New onset stroke	61.2 (44.4–77.9)	0.190	>1.728	92 (64.0–99.8)	42.2 (27.7–57.8)
Re-exploration for bleeding	60.1 (39.5–80.6)	0.336	>4.431	54 (25.1–80.8)	77.8 (62.9–88.8)
*de novo* dialysis	68.5 (38.8–98.2)	0.222	>15.18	40 (5.3–85.3)	98.1 (89.9–100.0)
Prolonged ventilation(≥72hr)	73.5 (57.7–89.3)	0.004	>1.547	100 (71.5–100.0)	40.4 (26.4–55.7)
ICU stay ≥7 days	63.1 (45.9–80.4)	0.135	>3.685	53 (27.8–77.0)	73.2 (57.1–85.8)
Hospital stay ≥30 days	78.2 (61.6–94.7)	0.001	>5.992	58 (27.7–84.8)	91.3 (79.2–97.6)

AUC, area under the curve; CI, confidence interval; ICU, intensive care unit.

# by Youden index.

According to the cutoffs of prolonged ventilation and a hospital stay of more than 30 days, we classified patients into 3 subgroups as follows: <1.547 (N = 18), 1.547–5.992 (N = 28), and > 5.992 ng/mL (N = 12). The results demonstrated that patients with higher lumican levels tended to experience poor outcomes for ventilation time, prolonged ventilation, length of ICU stay, and a hospital stay of more than 30 days (**S1 Table**).

Simultaneously, we compared the characteristics of patients with and without the primary outcome (prolonged ventilation). The results showed that patients with prolonged ventilation were more likely to be intubated before surgery, had longer bypass time, and were more likely to suffer from several in-hospital outcomes, including stroke, re-exploration for bleeding, prolonged ICU stay, prolonged hospital days, dialysis, and in-hospital mortality (**S2 Table**).

**Fig 2:**
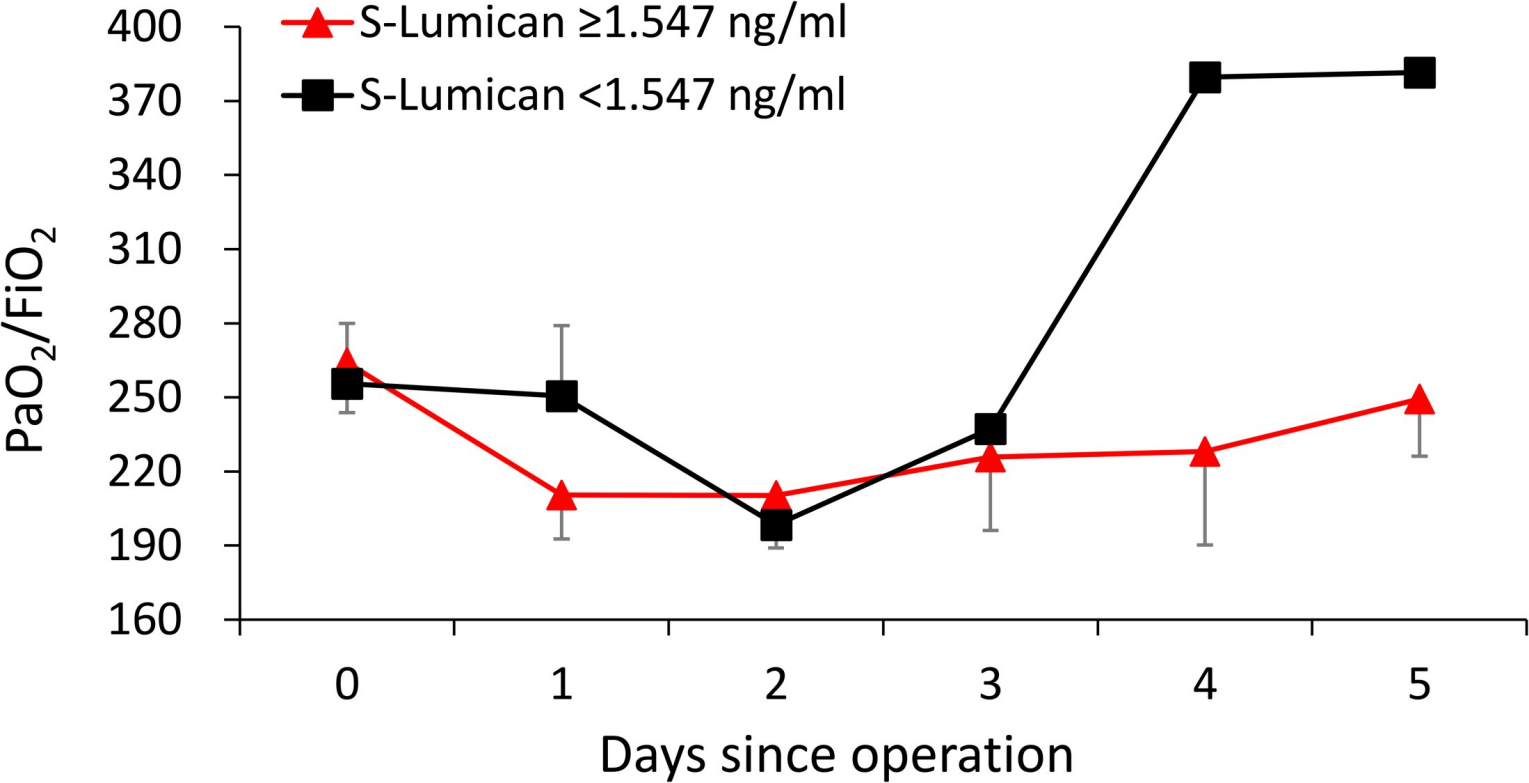
The mean and standard error of PaO_2_/FiO_2_ in patients with serum lumican levels of less than 1.547 ng/mL (n = 18) and those with serum lumican levels of greater than 1.547 ng/mL (n = 40). Abbreviation: PaO_2_ = partial pressure of arterial oxygen; FiO_2_ = percentage of inspired oxygen.

The daily oxygenation indices (PaO_2_/FiO_2_) are shown in **Fig 2**using 1.547 ng/mL as a cutoff. Although no statistical significance was reached, the group below the cutoff seemed to demonstrate an improvement on the fourth day after surgery, whereas the group above the cutoff displayed a stable low oxygenation index.

## Discussion

The current study demonstrated the potential ability of lumican to predict prolonged ventilation and hospital stay over 30 days. By using 1.547 ng/mL as a cutoff level, the group below the cutoff demonstrated an improvement in the oxygenation index on the fourth day after surgery, whereas the group above the cutoff level exhibited a low oxygenation index trend. To our knowledge, this is the first human study to investigate the association between the soluble lumican level and postoperative outcomes after aortic surgery. The results revealed that soluble lumican can be a potential biomarker for poor respiratory outcomes after aortic surgery. Therefore, this preliminary study directed the potential research area of lumican and poor respiratory outcomes in aortic diseases, and additional studies investigating the detailed mechanism and usefulness of soluble lumican are required.

### Lumican and aortic disease

Several studies have investigated the association between lumican and aortic disease. Gu et al. [23] first reported that the serum lumican level was significantly higher in patients with acute aortic dissection than in those with acute myocardial infarction and normal individuals. Therefore, they suggested that serum lumican can be a promising diagnostic biomarker for acute aortic dissection. The relationship of aortic dissection and the serum lumican level may be explained by the pathophysiological mechanism in aortic dissection^2^. Because of a tear in the aortic intima, blood flushes into the aortic wall, resulting in the formation of a false lumen. Hence, the serum lumican level is higher in patients with acute aortic dissection because lumican distributed in the aortic wall is released into circulation. Therefore, the serum lumican level can be a potential diagnostic biomarker; however, more studies are required to further evaluate its usefulness in diagnosing acute aortic dissection.

Harrison et al. [25] investigated potential biomarkers that predict future aneurysms in patients with bicuspid aortic valve receiving aortic valve surgery, and the results revealed that the serum lumican level demonstrated a low variance in the aneurysmal group but a high variance in the nonaneurysmal group. Therefore, they suggested that the serum lumican level is a potential biomarker for predicting future aneurysms in patients with bicuspid aortic valves. However, their study had a small sample size; therefore, the biomarker was not validated in the follow-up. Furthermore, the pathophysiological mechanism of lumican in bicuspid aortic valve and aneurysm formation is still unknown, and additional studies are warranted.

### Lumican and lung injury

Our animal study [22] demonstrated the role of lumican in the pathophysiological mechanism of ventilation-induced lung injury in mice. Mechanical ventilation upregulated the expression of lumican, which is involved in the fibrogenesis of lung injury through the extracellular signal-regulated kinase (ERK) 1/2 pathway. According to Gu’s studies [23,24], the serum lumican level was high in acute aortic dissection. The present study results demonstrated a high serum lumican level as a prognostic factor for prolonged ventilation and longer in-hospital stay. Our animal study [22] provided the pathophysiological basis of a high serum lumican level and poor respiratory outcomes. Taken together, a severe condition of aortic dissection may cause the release of more lumican into blood circulation. Moreover, lumican involves in fibrogenesis in lung injury; therefore, clinical data demonstrate prolonged ventilation use and longer in-hospital stay. Hence, serum lumican can be a potential biomarker for predicting poor postoperative outcomes. However, the detailed mechanism of aortic diseases and lung injury are still unclear, and more studies are required.

Our another study [26] reported that the incidence of acute respiratory distress syndrome (ARDS) after acute type A aortic dissection repair was 15.9%. The oxygenation index (PaO_2_/FiO_2_) of the ARDS group was lower than that of the non-ARDS group during the first 3 postoperative days and increased on the fourth postoperative day. This may explain why lumican level differentiated prolonged ventilation use of more than 3 days but not ICU stays of more than 7 days. That is, the influence of lung injury was more prominent during the first 3 postoperative days. Hence, by detecting postoperative serum lumican levels, doctors may be able to predict the patient’s respiratory outcomes. Those with a serum lumican level of >1.547 ng/mL may need prolonged mechanical ventilation use (over 3 days). By contrast, those with a serum lumican level below the cutoff value may tend to lean off the ventilation early. However, the role of lumican in lung injury and recovery after aortic surgery remains unknown, and more studies are required to investigate the detailed mechanisms.

### Clinical implications

The present study revealed that the optimal cutoff level for prolonged ventilation was 1.547 ng/mL, with a sensitivity of 100%. Therefore, lumican level may be an effective biomarker for intensive care staff to predict prolonged ventilation upon patients’ arrival at the ICU after aortic surgery. When the lumican level is below 1.547 ng/mL, a fair respiratory outcome in the patient is a reasonable prediction; additionally, weaning from the ventilator can be applied shortly thereafter. However, when the lumican level is above 1.547 ng/mL, more aggressive respiratory care such as fluid limitation and limited blood transfusion may be required. Furthermore, for patients whose lumican level is above 5.992 ng/mL, in-hospital stay of more than 30 days can be expected, with a specificity of 91.3%. This may occur because a higher lumican level indicates an aortic disease of greater severity; therefore, additional complications may occur, resulting in a prolonged in-hospital stay. For such patients, medical staff may need to monitor clinical conditions more closely. Early interventions may also be warranted.

### Study limitations

The study is limited by a small sample size and could not investigate different conditions in aortic dissection and aneurysm. Additionally, the limited sample size and the small number of events caused difficulty in applying multivariable adjustments. Furthermore, baseline and serial lumican levels were not involved in this study, so the influence of preoperative lumican levels and the serial change could not be evaluated. Simultaneously, the pathophysiological mechanisms of different aortic diseases and the role of the lumican levels are still unclear. Therefore, more studies are warranted to investigate the involvement of lumican in the pathophysiological mechanisms of aortic diseases and the usefulness of lumican to be a promising biomarker in predicting perioperative outcomes in aortic surgery.

## Conclusion

This preliminary study demonstrated that the serum lumican level was associated with prolonged ventilation and longer in-hospital stay for patients receiving aortic surgery. Therefore, the serum soluble lumican level may be a potential prognostic factor for predicting poor postoperative outcomes after aortic surgery. However, the detailed biomolecular mechanisms are still unclear, and more studies are warranted in the future.

## Supporting information

S1 Table In-hospital outcomes of patients stratified by lumican level. (DOCX)

S2 Table Demographical, preoperative, and perioperative data of study patients according to the presence or absence of primary outcome. (DOCX)
