# Supplementary material for: Level of serum soluble lumican and risks of perioperative complications in patients receiving aortic surgery
Source: PLoS One. 2021 Mar 4;16(3):e0247340. doi: 10.1371/journal.pone.0247340 (PMC7932520; doi:10.1371/journal.pone.0247340)
Supplement: S1 Table — (DOCX) [file pone.0247340.s001.docx]

**Supplemental Table 1.** In-hospital outcomes of patients stratified by lumican level

|  | Lumican, ng/mL | | |  |
| --- | --- | --- | --- | --- |
| Variable | <1.547  (*n* = 18) | 1.547-5.992  (*n* = 28) | >5.992  (*n* = 12) | *P* for trend |
| Ventilation time, hours | 16.3 [5.8, 21.0] | 19.4 [8.0, 68.1] | 48.8 [20.8, 236.9] | 0.011 |
| Prolonged ventilation (≥72 hrs.) | 0 (0.0) | 6 (21.4) | 5 (41.7) | 0.004 |
| Cardiogenic shock and need MCS | 0 (0.0) | 0 (0.0) | 1 (8.3) | 0.121 |
| New onset stroke | 1 (5.6) | 8 (28.6) | 4 (33.3) | 0.057 |
| Re-exploration for bleeding | 4 (22.2) | 4 (14.3) | 5 (41.7) | 0.304 |
| ICU stay, days | 3.0 [2.0, 4.0] | 4.0 [2.5, 7.5] | 6.0 [3.5, 18.0] | 0.002 |
| ICU stay ≥7 days | 3 (16.7) | 8 (28.6) | 6 (50.0) | 0.056 |
| Hospital stay, days | 14.5 [11.0, 19.0] | 15.5 [8.5, 22.5] | 33.5 [9.0, 50.0] | 0.125 |
| Hospital stay ≥30 days | 1 (5.6) | 4 (14.3) | 7 (58.3) | 0.001 |
| *de novo* dialysis | 1 (5.6) | 2 (7.1) | 2 (16.7) | 0.323 |
| Sepsis | 1 (5.6) | 1 (3.6) | 0 (0.0) | 0.427 |
| Deep wound infection | 0 (0.0) | 2 (7.1) | 0 (0.0) | 0.836 |
| Mortality | 0 (0.0) | 2 (7.1) | 0 (0.0) | 0.836 |

MCS, mechanical circulation support; ICU, intensive care unit.
