# Supplementary material for: Level of serum soluble lumican and risks of perioperative complications in patients receiving aortic surgery
Source: PLoS One. 2021 Mar 4;16(3):e0247340. doi: 10.1371/journal.pone.0247340 (PMC7932520; doi:10.1371/journal.pone.0247340)
Supplement: S2 Table — (DOCX) [file pone.0247340.s002.docx]

**Supplemental Table 2.** Demographical, preoperative, and perioperative data of study patients according to the presence or absence of primary outcome

|  | Prolonged ventilation (≥72 hrs.) | |  |
| --- | --- | --- | --- |
| Variable | Yes (*n* = 11) | No (*n* = 47) | *P* |
| Demographics |  |  |  |
| Age, years | 59.0 ± 9.5 | 55.2 ± 14.3 | 0.409 |
| Female | 7 (63.6) | 35 (74.5) | 0.475 |
| Body mass index (kg/m^2^) | 28.8 ± 5.2 | 27.1 ± 5.7 | 0.405 |
| Smoking | 3 (27.3) | 13 (27.7) | 1.000 |
| Comorbidity |  |  |  |
| Heart failure | 0 (0.0) | 8 (17.0) | 0.331 |
| Diabetes mellitus | 0 (0.0) | 2 (4.3) | 1.000 |
| Hypertension | 8 (72.7) | 35 (74.5) | 1.000 |
| Old stroke | 2 (18.2) | 2 (4.3) | 0.159 |
| COPD | 0 (0.0) | 1 (2.1) | 1.000 |
| Chronic kidney disease | 2 (18.2) | 4 (8.5) | 0.318 |
| Surgical type |  |  |  |
| Ascending aorta replacement | 3 (27.3) | 20 (42.6) | 0.499 |
| Aortic arch replacement | 7 (63.6) | 20 (42.6) | 0.315 |
| Aortic root replacement (Bentall operation) | 1 (9.1) | 8 (17.0) | 1.000 |
| Elephant trunk | 2 (18.2) | 10 (21.3) | 1.000 |
| Additional surgery |  |  |  |
| CABG | 1 (9.1) | 2 (4.3) | 0.474 |
| Valve surgery | 1 (9.1) | 10 (21.3) | 0.671 |
| Intra-operative data, min |  |  |  |
| Bypass time, min | 328 [213, 385] | 219 [168, 272] | 0.011 |
| Clamp time, min | 152 [136, 200] | 144 [125, 171] | 0.134 |
| Arrest time, min | 45 [38, 50] | 33 [0, 53] | 0.160 |
| HTK solution | 11 (100.0) | 43 (91.5) | 1.000 |
| Preoperative status |  |  |  |
| Hemopericardium | 4 (36.4) | 10 (21.3) | 0.433 |
| Intubation | 2 (18.2) | 0 (0.0) | 0.033 |
| Neurological defect | 0 (0.0) | 8 (17.0) | 0.331 |
| Moderate or severe aortic regurgitation | 4 (36.4) | 19 (40.4) | 1.000 |
| In-hospital outcome |  |  |  |
| Ventilation time, hours | 192 [125, 638] | 14.8 [6.5, 36.5] | <0.001 |
| Cardiogenic shock and need MCS | 1 (9.1) | 0 (0.0) | 0.190 |
| New onset stroke | 7 (63.6) | 6 (12.8) | 0.001 |
| Re-exploration for bleeding | 6 (54.5) | 7 (14.9) | 0.010 |
| ICU stay, days | 13 [10, 24] | 3 [2, 4] | <0.001 |
| ICU stay ≥7 days | 10 (90.9) | 7 (14.9) | <0.001 |
| Hospital stay, days | 40 [15, 66] | 14 [8, 21] | 0.002 |
| Hospital stay ≥30 days | 6 (54.5) | 6 (12.8) | 0.006 |
| *de novo* dialysis | 3 (27.3) | 2 (4.3) | 0.042 |
| Sepsis | 1 (9.1) | 1 (2.1) | 0.346 |
| Deep wound infection | 1 (9.1) | 1 (2.1) | 0.346 |
| Mortality | 2 (18.2) | 0 (0.0) | 0.033 |
| Lumican, ng/ml | 4.6 [2.3, 22.0] | 2.3 [1.2, 4.4] | 0.016 |

CABG, coronary artery bypass graft; COPD, chronic obstructive pulmonary disease; MCS, mechanical circulation support; ICU, intensive care unit.

Data were presented as frequency (percentage) or mean ± standard deviation or median [Quartile 1, Quartile 3].
